# Anti-proliferative effects of *Salacia reticulata* leaves hot-water extract on interleukin-1β-activated cells derived from the synovium of rheumatoid arthritis model mice

**DOI:** 10.1186/1756-0500-5-198

**Published:** 2012-04-26

**Authors:** Yuusuke Sekiguchi, Hiroshi Mano, Sachie Nakatani, Jun Shimizu, Kenji Kobata, Masahiro Wada

**Affiliations:** 1 Department of Clinical Dietetics and Human Nutrition, Faculty of Pharmaceutical Sciences, Josai University, 1-1 Keyakidai, Sakado, Saitama, 350-0295, Japan

## Abstract

**Background:**

*Salacia reticulata* (SR) is a plant native to Sri Lanka. In ayurvedic medicine, SR bark preparations, taken orally, are considered effective in the treatment of rheumatism and diabetes. We investigated the ability of SR leaves (SRL) to inhibit *in vitro* the interleukin-1β (IL-1β)-activated proliferation of synoviocyte-like cells derived from rheumatoid arthritis model mice.

**Findings:**

Inflammatory synovial tissues were harvested from type II collagen antibody-induced arthritic mice. From these tissues, a synoviocyte-like cell line was established and named MTS-C H7. To determine whether SRL can suppress cell proliferation and gene expression in MTS-C H7 cells, fractionation of the SRL hot-water extract was performed by high-performance liquid chromatography (HPLC), liquid-liquid extraction, sodium dodecyl sulphate-polyacrylamide gel electrophoresis (SDS-PAGE), and protease digestion.

The 50% inhibitory concentration of the SRL hot-water extract against MTS-C H7 cells proliferation was ~850 μg/mL. Treatment with a low dose (25 μg dry matter per millilitre) of the extract inhibited IL-1β-induced cell proliferation and suppressed the expression of the matrix metalloproteinase (MMP) genes in MTS-C H7 cells. Various polyphenolic fractions obtained from HPLC and the fractions from liquid-liquid extraction did not affect cell proliferation. Only the residual water sample from liquid-liquid extraction significantly affected cell proliferation and the expression of MMP genes. The results of SDS-PAGE and protease digestion experiment showed that low molecular weight proteins present in SRL inhibited the IL-1β-activated cell proliferation.

**Conclusions:**

We surmised that the residual water fraction of the SRL extract was involved in the inhibition of IL-1β-activated cell proliferation and regulation of mRNA expression in MTS-C H7 cells. In addition, we believe that the active ingredients in the extract are low molecular weight proteins.

## Findings

*Salacia reticulata* (SR) is a plant native to Sri Lanka. In traditional Sri Lankan medicine, called ‘Ayurveda’, the roots and stems of SR are used for the prevention of rheumatism and diabetes [1,2]. For example, the roots and stems of SR are known to contain unique compounds such as salacinol, kotalanol, and mangiferin [3-5]. In a previous study with a murine disease model, SR leaves (SRL) ameliorated the symptoms of rheumatoid arthritis (RA) [6].

Although the aetiology of RA is not yet fully understood, classical studies have suggested that autoantibody production, inflammatory cell infiltration, and tumour-like proliferation of synovial ‘pannus’ are involved in the pathogenesis of RA [7,8]. Recent research on the roles of fibroblast-like synoviocytes in the pannus has gained recognition [9]. The pannus releases several proinflammatory mediators and matrix metalloproteinases [10]. RA treatment is currently based mainly on the administration of anti-inflammatory drugs and anti-rheumatic drugs [11].

In this study, we investigated the potential of SRL as a prophylactic or therapeutic agent for RA.

### Preparation of samples

SRL were sun-dried. After removing the damaged leaves, the dried intact leaves were pulverized in a food mill and filtered through a 150-μm mesh sieve to obtain the powder. The powder was boiled in pure distilled water, and the extract solution was centrifuged for 20 min at 2190 × *g* to remove the pellet. The supernatant was filtered through a 0.20-μm filter membrane and dried using a freeze dryer. We purchased commercial agents prepared from stems of SR, such as mangiferin (Sigma-Aldrich Co., USA), triptotriterpenic acid B (AApin Chemicals Abingdon, UK), and (−)-epicatechin (Sigma-Aldrich Co., USA). These agents were dissolved in ethanol. All samples were stored at −20°C until use.

### Cell culture

Collagen antibody-induced arthritic (CAIA) mice were generated using DBA/1 J mice as reported previously [8]. Synovial tissues were obtained from the knee joints of these mice. The inflammatory synovial tissues were minced and stirred with type IV collagenase (Sigma-Aldrich Co., USA) in serum-free DMEM/F12 medium at 37°C for 3 h in an incubator shaker. The synovial tissue lysate was then filtered through a 40-μm nylon mesh, washed extensively, and seeded at 1 cell/well in 96-well microplates. The cells were cultured in DMEM/F12 supplemented with 10% FBS and benzylpenicillin potassium (100 units/mL) at 37°C/5% CO_2_. The inflammatory synovial tissue has been reported that the gene of Synoviolin is over-expressed [12]. Therefore, the cell named MTS-C H7 of high-expressed genes of Synoviolin was established. The study was performed in accordance with the National Institutes of Health Guide for the Care and Use of Laboratory Animals and the Institutional Animal Care and Use Committee of Josai University, Saitama, Japan.

### Cell viability assay

MTS-C H7 cells were plated in 96-well microplates at a density of 5 × 10^3^/well. After 3 h, the SRL extract was added and cultured for 24 h. After incubattion, 10 μL of the cell proliferation reagent WST-1 (Roche Diagnostics, USA) was added and incubated for 30 min. Cell proliferation was measured at 450 nm by using a spectrophotometer.

### Cell proliferation assay

MTS-C H7 cells were plated on 96-well microplates at a density of 5 × 10^3^/well. After 3 h, mouse IL-1β or other samples were added and cultured for 24 h. The cell proliferation assay was performed as describe above.

### HPLC analysis

The HPLC system consisted of an LC-Organizer, L-6200 Intelligent pump, L-4200H UV–VIS Detector, and ELITE LaChrom Column Oven L-2350 (HITACHI, Japan). Separation was carried out on a Wakosil-II 5C18AR column (250 mm × 4.6 mm, 5 μm). HPLC conditions were performed according to the reference [13]. All samples were evaporated and dried, dissolved in ethanol. All samples were stored at −20°C until use.

### Liquid-liquid distribution assay

The SRL extract was separated by hexane, diethyl ether, ethyl acetate, and *n*-butanol. The up layers and residual water fraction were collected. These samples were evaporated and dried, dissolved in ethanol or ultrapure water. All samples were stored at −20°C until use.

### RNA extraction and reverse transcription (RT)-PCR

MTS-C H7 cells were cultured at approximately 2 × 10^5^/dish. After 24 h, mouse IL-1β and other samples were added and cultured for 24 h. RNA was extracted using the TRIzol reagent according to the manufacturer’s instructions. The term of genes expression has been reported previously [6].

### SDS-PAGE

SDS-PAGE was used to determine the molecular weight and purity of the protein isolated from SRL. The samples were run on a MULTIGEL Mini 15/25 (COSMO BIO, Tokyo, Japan) gradient gel with the SDS-PAGE buffer (0.1% SDS, 0.05 M Tris, 0.05 M tricine). After electrophoresis, the gel was fixed with ultrapure water for 15 min and stained with GelCode Blue Safe Protein Stain (Thermo Fisher Scientific K.K., Waltham, USA) for 1 h.

### Protease digestion assay

SRL extract was mixed with CPaseY incubated at 25°C from 0 to 60 min. MTS-C H7 cells were plated in 96-well microplates at a density of 5 × 10^3^/well. After 3 h, SRL + CPaseY and IL-1β were added and cultured for 24 h. The cell proliferation assay using samples were performed as describe above.

### Statistical analysis

The results are expressed as the mean and standard deviation (SD) of three independent experiments. Statistical analysis was carried out with Stat-Mate III Version 3.18 (ATMS Co., Ltd., Japan). Data distributions were compared using the Analysis of variance.

### Cytotoxic effect of SRL on MTS-C H7 cells

**Figure 1:**
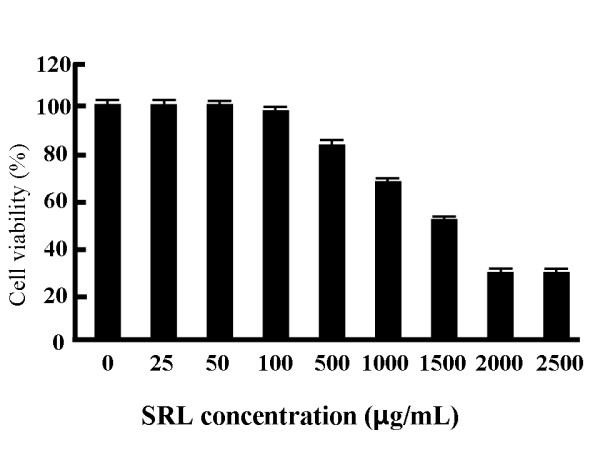
**Cytotoxic activity of the SRL hot-water extract against MTS-C H7 cells.** MTS-C H7 cells were treated with the SRL extract at concentrations of 25, 50, 100, 500, 1000, 1500, 2000, and 25,00 μg dry matter/mL for 24 h. The results are expressed as mean (SD), n = 3. The experiment was performed in triplicate.

As shown in Figure 1, cell viability was not changed by 25 and 100 μg dry matter/mL; however, it was decreased by 500 μg dry matter/mL and showed to decreased until 2000 μg dry matter/mL. We calculated the IC_50_ according to the literature [14] and obtained the value of ~850 μg/mL.

### Effect of SRL on MTS-C H7 cell proliferation induced by inflammatory mediators

**Figure 2:**
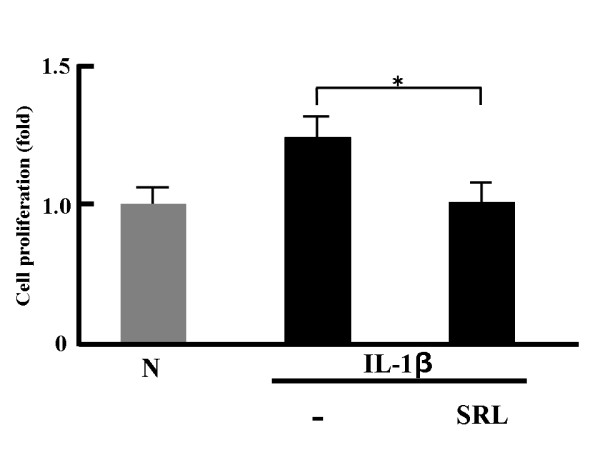
**Anti-proliferative effects of the SRL hot-water extract on MTS-C H7 cells.** MTS-C H7 cells were treated with mouse IL-1β (10 ng/mL) and SRL extract (50 μg dry matter/mL) for 24 h. The results are expressed as mean (SD), n = 3. Significantly different from IL-1β: * *p* < 0.05. The experiment was performed in triplicate.

As shown in Figure 2, MTS-C H7 cell proliferation in the presence of the SRL was significantly decreased compared with only IL-1β. Thus, the SRL clearly inhibits IL-1β-induced cell proliferation.

### Effects of reference compounds present in the stems of SR on cell proliferation

**Figure 3:**
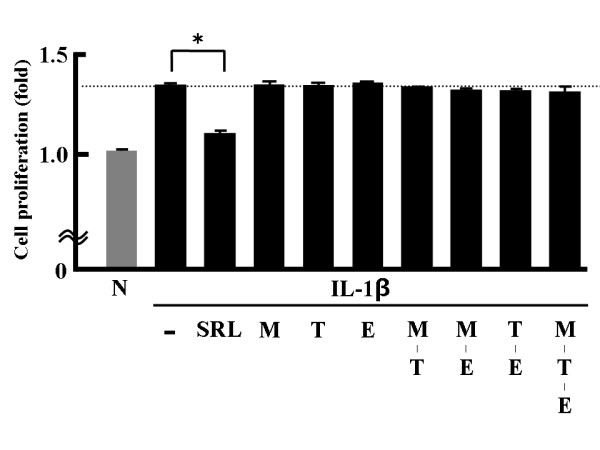
**Effects of commercially available compounds present in*Salacia reticulata*roots and stems on MTS-C H7 cell proliferation.** MTS-C H7 cells were treated for 24 h with 10 ng/mL mouse IL-1β, SRL at 50 μg dry matter/mL, 10^−8^ M mangiferin (M), 10^−8^ M triptotriterpenic acid B (T), or 10^−8^ M (−)-epicatechin (E). Cell proliferation was assessed by the WST-1 assay. The results are expressed as mean (SD), n = 3. Significantly different from IL-1β: * *p* < 0.05. The experiment was performed in triplicate.

As shown in Figure 3, M, T, or E did not affect IL-1β-activated cell proliferation, which indicates that the 3 tested reagents are not among the active ingredients of SR.

### HPLC analysis of the SRL hot-water extract

**Figure 4:**
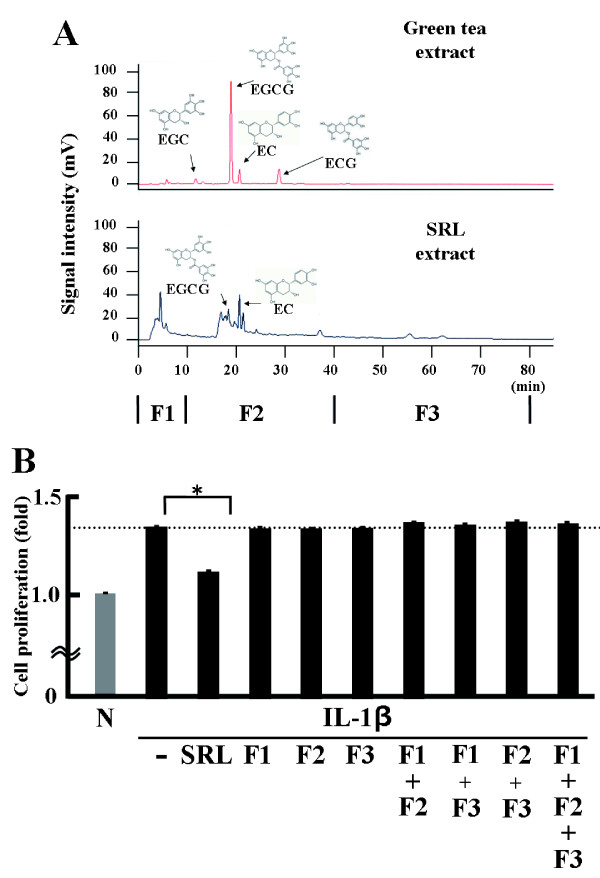
**Effects of HPLC samples of the fractionated SRL hot-water extract on MTS-C H7 cell proliferation. (A)** Major catechins, i.e., (−)-epicatechin (EC), (−)-epicatechin gallate (ECG), (−)-epigallocatechin (EGC), and (−)-epigallocatechin gallate (EGCG), in green tea extract and SRL extract. Fraction 1 (F1): 100% solvent A, 0–10.0 min; fraction 2 (F2): 90% solvent A + 10% solvent B, 10.1–40.0 min; and fraction 3 (F3): 40.1–80.0 min. **(B)** Anti-proliferative effects of the fractionated samples of the SRL extract on the growth of MTS-C H7 cells after 24-h treatment. MTS-C H7 cells were treated for 24 h with mouse IL-1β (10 ng/mL); SRL extract (25 μg dry matter/mL); or F1, F2, and F3 (25 μg dry matter weight/mL). Cell proliferation was assessed by the WST-1 assay. The results are expressed as mean (SD), n = 3. Significantly different from IL-1β: * *p* < 0.05. The experiment was performed in triplicate.

The HPLC analysis data for 2 different extracts are shown in Figure 4A. The (−)-epicatechin (EC), epicatechin gallate, epigallocatechin, and epigallocatechin gallate (EGCG) catechins were detected in a green tea extract used as a catechin control. The EC and EGCG were detected in the SRL extract. These results show that the components of the hot-water extract from *Camellia sinensis* (Japanese green tea) leaves are different from those in the SRL extract.

### Effects of various HPLC fractions of the SRL extract on cell proliferation

As shown in Figure 4B, Cell proliferation in the presence of IL-1β did not change when F1, F2, or F3 were added to the culture. These results show that none of the HPLC polyphenolic fractions of the SRL extract suppressed the IL-1β-induced cell proliferation.

### Effects of various liquid-liquid distribution fractions of the SRL extract on cell proliferation

**Figure 5:**
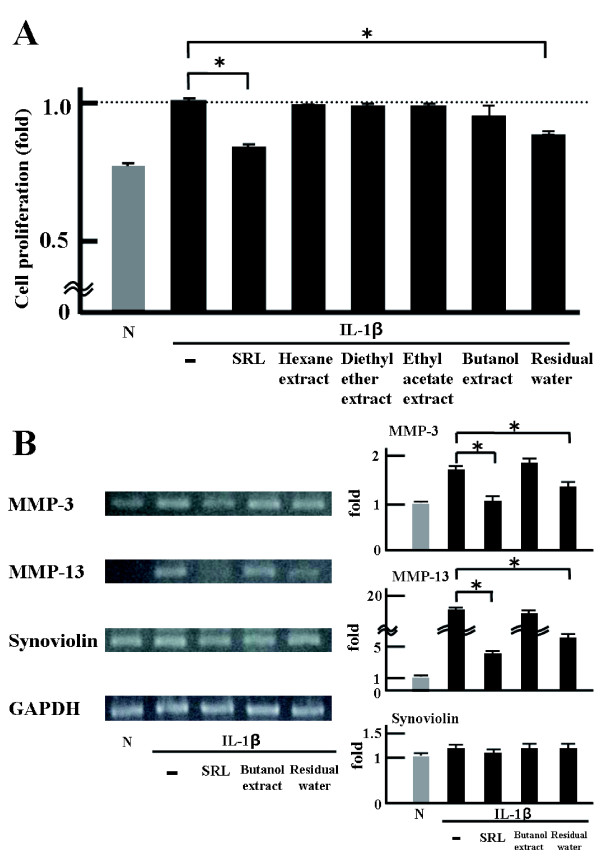
**Effects of the fractionated SRL hot-water extract samples on cell proliferation and mRNA expression. (A)** Anti-proliferative effects of SRL extract samples fractionated according to the polarity of constituents on the growth of MTS-C H7 cells after 24-h treatment. MTS-C H7 cells were treated with mouse IL-1β (10 ng/mL); SRL (50 μg dry matter/mL); hexane, diethyl ether, ethyl acetate, and *n*-butanol extract samples (all 50 μg dry matter/mL); or residual water sample (50 μg dry matter/mL). Cell proliferation was assessed by the WST-1 assay. Results are expressed as mean (SD), n = 3. Significantly different from IL-1β: * *p* < 0.05. The experiment was performed in triplicate. **(B)** MTS-C H7 cells were treated with mouse IL-1β (10 ng/mL), SRL (50 μg dry matter/mL), *n*-butanol extract sample (50 μg dry matter/mL), or residual water sample (50 μg dry matter/mL). Gene expression was detected by RT-PCR. Results are expressed as mean (SD), n = 3. Significantly different from IL-1β: * *p* < 0.05. The experiment was performed in triplicate.

As shown in Figure 5A, cell proliferation in the presence of the residual water sample was decreased compared with IL-1β alone. These results show that the water-soluble component in the SRL extract attenuated the IL-1β-induced cell proliferation.

### Effect of residual water from the SRL hot-water extract on gene expression results

As shown in Figure 5B, SRL extract and residual water samples were significant decrease in the mRNA levels for *MMP-3* and *MMP-13*. These results show the possibility that the active ingredients in the residual water fraction decrease the activation of gene expression by IL-1β.

### Characterization of the active ingredients as peptides

**Figure 6:**
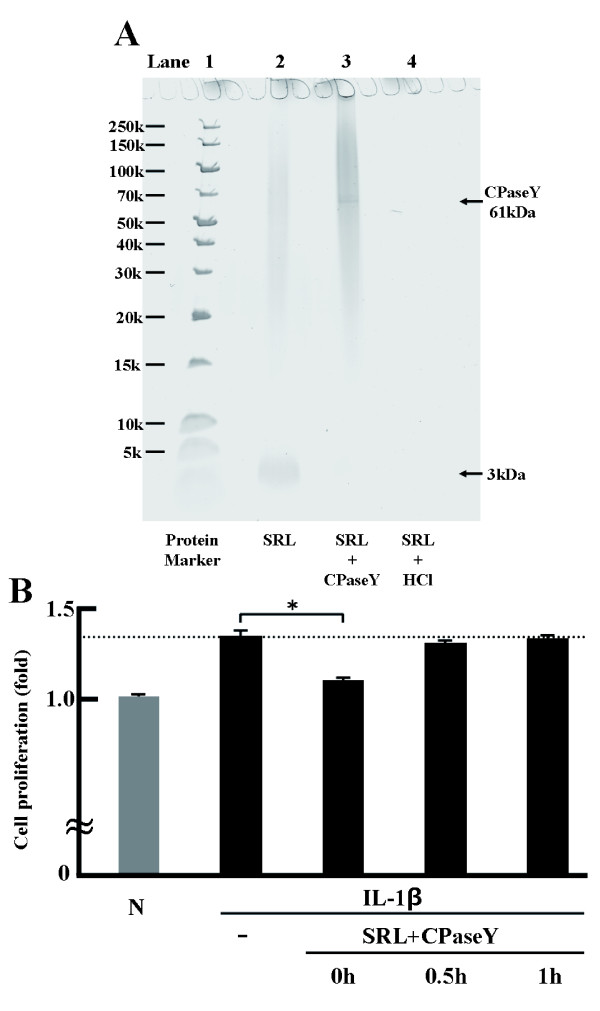
**Characterization of the active ingredients as peptides.** SRL extract (0.5 mg dry matter) was mixed with carboxypeptidase Y (CPaseY) (2.38 units) and incubated at 25°C for 24 h. After incubation, the samples were heated at 95°C for 5 min. SRL extract was mixed with HCl (6 mol/L) and heated at 90°C for 2 h. **(A)** The SRL-only sample was subjected to electrophoresis (lane 2). SRL was treated with CPaseY (lane 3). SRL was treated HCl (lane 4). Proteins were detected by SDS-PAGE with gels stained with GelCode Blue Safe Protein Stain. **(B)** MTS-C H7 cells were treated with mouse IL-1β (10 ng/mL) and SRL treated with CPaseY. Cell proliferation was assessed by the WST-1 assay. The results are expressed as mean (SD), n = 3. Significantly different from IL-1β: * *p* < 0.05. The experiment was performed in triplicate.

As shown in Figure 6A, using only the SRL extract, a band of ~3 kDa was observed. In addition, in the sample treated with HCl, no band could be observed at any position. As shown in Figure 6B, at MTS-C H7 cell proliferation in the presence of CPaseY-treated SRL extract was not decreased compared with only IL-1β. Moreover,HCl-treated SRL extract showed a similar results (data not shown).

## Discussion and Conclusions

RA treatments often cause severe adverse effects depending on the patient’s sensitivity or the drug dosage [15-17]. Traditional herbal therapy for RA takes advantage of using extracts of plants [18,19]. However, herbal medicines have been reported to cause fewer adverse effects [20,21].

The present study contributes to elucidating the effect of the SRL extract and residual water fraction sample on IL-1β-activated cell proliferation and gene expression against MTS-C H7 cells. The amount of protein in SRL extract (100 mg dry matter/ml) was confirmed by Lowry method to be 0.52 mg/mL [22]. Moreover, SDS-PAGE analysis confirmed that SRL contains low molecular weight proteins (≤3 kDa).

In conclusion, we investigated whether the SRL can affect the functions of IL-1β-activated MTS-C H7 cells. Our results show that the active ingredients might be 3 kDa peptides. Moreover, it is well known that salacinol and kotalanol are similar in structure. Salacinol was reported to be in *n*-butanol fraction [23]. The active ingredients did not appear to be catechins, salacinol, kotalanol, or mangiferin.

SRL appears to have potential as a functional food or herbal medicine for RA. In order to identify the sequence of these peptides, the future, we need to consider how to purify the peptides.

## Competing interests

The authors declare that they have no competing interests.

## Authors’ contributions

YS conducted all the experiments and wrote the manuscript. HM developed all of the experiments performed in this study and critically read the manuscript and offered useful suggestions for improvement. SN, JS, KK and MW helped to draft the manuscript. All authors read and approved the final manuscript.

## References

[B1] Shimada T Nagai E Harasawa Y Akase T Aburada T Iizuka S Miyamoto K Aburada M Metabolic disease prevention and suppression of fat accumulation by Salacia reticulata J Nat Med 2010 64 266 274 10.1007/s11418-010-0401-1 20225078

[B2] Sim L Jayakanthan K Mohan S Nasi R Johnston BD Pinto BM Rose DR New glucosidase inhibitors from an Ayurvedic herbal treatment for type 2 diabetes: structures and inhibition of human intestinal maltase-glucoamylase with compounds from Salacia reticulata Biochemistry 2010 49 443 451 10.1021/bi9016457 20039683

[B3] Yoshikawa M Morikawa T Matsuda H Tanabe G Muraoka O Absolute stereostructure of potent alpha-glucosidase inhibitor, salacinol, with unique thiosugar sulfonium sulfate inner salt structure from Salacia reticulata Bioorg Med Chem 2002 10 1547 1554 10.1016/S0968-0896(01)00422-9 11886816

[B4] Yoshikawa M Murakami T Yashiro K Matsuda H Kotalanol, a potent alpha-glucosidase inhibitor with thiosugar sulfonium sulfate structure, from antidiabetic Ayurvedic medicine Salacia reticulata Chem Pharm Bull (Tokyo) 1998 46 1339 1340 10.1248/cpb.46.1339 9734318

[B5] Im R Mano H Matsuura T Nakatani S Shimizu J Wada M Mechanisms of blood glucose-lowering effect of aqueous extract from stems of Kothala himbutu (Salacia reticulata) in the mouse J Ethnopharmacol 2009 121 234 240 10.1016/j.jep.2008.10.026 19028559

[B6] Sekiguchi Y Mano H Nakatani S Shimizu J Wada M Effects of the Sri Lankan medicinal plant, Salacia reticulata, in rheumatoid arthritis Genes Nutr 2010 5 89 96 10.1007/s12263-009-0144-3 19727885 PMC2820195

[B7] Funk JL Cordaro L Wei H Benjamin JB Yocum DE Synovium as a source of increased amino-terminal parathyroid hormone-related protein expression in rheumatoid arthritis. A possible role for locally produced parathyroid hormone-related protein in the pathogenesis of rheumatoid arthritis J Clin Invest 1998 101 1362 1371 9525978 10.1172/JCI484 PMC508713

[B8] Parekh RB Dwek RA Sutton BJ Fernandes DL Leung A Stanworth D Rademacher TW Mizuochi T Taniguchi T Matsuta K Association of rheumatoid arthritis and primary osteoarthritis with changes in the glycosylation pattern of total serum IgG Nature 1985 316 452 457 10.1038/316452a0 3927174

[B9] Seki M Sakata KM Oomizu S Arikawa T Sakata A Ueno M Nobumoto A Niki T Saita N Ito K Dai SY Katoh S Nishi N Tsukano M Ishikawa K Yamauchi A Kuchroo V Hirashima M Beneficial effect of galectin 9 on rheumatoid arthritis by induction of apoptosis of synovial fibroblasts Arthritis Rheum 2007 56 3968 3976 10.1002/art.23076 18050192

[B10] Woolley DE Tetlow L Mast cell activation and its relation to proinflammatory cytokine production in the rheumatoid lesion Arthritis Res 2000 2 65 74 10.1186/ar70 11219391 PMC17805

[B11] van Tuyl LH Lems WF Voskuyl AE Kerstens PJ Garnero P Dijkmans BA Boers M Tight control and intensified COBRA combination treatment in early rheumatoid arthritis: 90% remission in a pilot trial Ann Rheum Dis 2008 67 1574 1577 18625629 10.1136/ard.2008.090712

[B12] Amano T Yamasaki S Yagishita N Tsuchimochi K Shin H Kawahara K Aratani S Fujita H Zhang L Ikeda R Fujii R Miura N Komiya S Nishioka K Maruyama I Fukamizu A Nakajima T Synoviolin/Hrd1, an E3 ubiquitin ligase, as a novel pathogenic factor for arthropathy Genes Dev 2003 17 2436 2449 10.1101/gad.1096603 12975321 PMC218080

[B13] Lin J-K Lin Chih-Li Liang Yu-Chih Lin-Shiau Shoei-Yn Juan I-Ming Survey of catechins, gallic acid, and methylxanthines in green, oolong, pu-erh, and black teas J. Agric. Food Chem 1998 46 3635 3642 10.1021/jf980223x

[B14] Mahavorasirikul W Viyanant V Chaijaroenkul W Itharat A Na-Bangchang K Cytotoxic activity of Thai medicinal plants against human cholangiocarcinoma, laryngeal and hepatocarcinoma cells in vitro BMC Complement Altern Med 2010 10 55 10.1186/1472-6882-10-55 20920194 PMC2956707

[B15] Maeda-Hagiwara M Watanabe K Aggravating effect of ergometrine on pyloric antral lesions in indomethacin-treated animals and stimulating effect of this drug on gastric secretion Jpn J Pharmacol 1981 31 891 896 10.1254/jjp.31.891 7334734

[B16] Thamer M Hernán MA Zhang Y Cotter D Petri M Prednisone, lupus activity, and permanent organ damage J Rheumatol 2009 36 560 564 10.3899/jrheum.080828 19208608 PMC3624968

[B17] Chikura B Sathi N Lane S Dawson JK Variation of immunological response in methotrexate-induced pneumonitis Rheumatology 2008 47 1647 1650 10.1093/rheumatology/ken356 18812430

[B18] Mur E Hartig F Eibl G Schirmer M Randomized double blind trial of an extract from the pentacyclic alkaloid-chemotype of Uncaria tomentosa for the treatment of rheumatoid arthritis J Rheumatol 2002 29 678 681 11950006

[B19] Tao X Cai J Lipsky PE The identity of immunosuppressive components of the ethyl acetate extract and chloroform methanol extract (T2) of Tripterygium wilfordii Hook F J Pharmacol Exp Ther 1995 272 1305 1312 7891348

[B20] López Galera RM Ribera Pascuet E Esteban Mur JI Montoro Ronsano JB Juárez Giménez JC Interaction between cat’s claw and protease inhibitors atazanavir, ritonavir and saquinavir Eur J Clin Pharmacol 2008 64 1235 1236 10.1007/s00228-008-0551-1 18712519

[B21] Lopez LM Grimes DA Schulz KF Nonhormonal drugs for contraception in men: a systematic review Obstet Gynecol Surv 2005 60 746 752 10.1097/01.ogx.0000182905.71077.13 16250923

[B22] Audo C Barbara J Chabane H Armange M Leynadier F The en 455–3 modified lowry assay does not yield a reliable estimate of the allergenicity level of latex gloves with low total protein content Med Sci Monit 2004 10 181 186 15232516

[B23] Girón MD Sevillano N Salto R Haidour A Manzano M Jiménez ML Rueda R López-Pedrosa JM Salacia oblonga extract increases glucose transporter 4-mediated glucose uptake in L6 rat myotubes: role of mangiferin Clin Nutr 2009 28 565 574 10.1016/j.clnu.2009.04.018 19477051

